# Long-Term Application of Fermented Fertilizer Attenuates the Accumulation of Antibiotic Resistance Genes in Aquaculture Sediment

**DOI:** 10.3390/microorganisms14061193

**Published:** 2026-05-25

**Authors:** Zhijing Yang, Wentao Xv, Yingchun Cai, Hailong Gu, Yaming Feng

**Affiliations:** Taizhou Institute of Agricultural Sciences, Jiangsu Academy of Agricultural Sciences, Taizhou 225300, China; 20162203@jaas.ac.cn (Z.Y.); xuwentao@jaas.ac.cn (W.X.); 20142201@jaas.ac.cn (Y.C.)

**Keywords:** fertilizer, aquaculture, antibiotic resistance genes, microbial community, mobile genetic element

## Abstract

Aquaculture sediments are increasingly recognized as important reservoirs of antibiotic resistance genes (ARGs). Although thermophilic fermentation is widely used to reduce ARGs and pathogens in manure, most biosafety assessments stop at the fertilizer product itself, leaving unresolved whether these benefits persist after application to aquaculture sediments. Here, we compared inorganic fertilizer (IF), raw manure (RM), and fermented fertilizer (FF) to test whether fermentation confers sustained biosafety benefits in aquaculture pond sediments. After a 6-month co-culture period, sediment samples were analyzed using shotgun metagenomic sequencing, ARG and mobile genetic element (MGE) profiling, antibiotic residue determination, and network analyses. Long-term fertilization significantly altered sediment physicochemical properties, microbial community composition, and resistome structure. Among the three groups, the RM exhibited the highest total ARG abundance and the greatest number of unique ARG subtypes, with significant enrichment of multidrug resistance genes as well as pathogen-, disease-, and host-associated mobile genetic elements (MGEs). In contrast, the FF group showed the lowest total ARG abundance and fewest unique ARG subtypes, along with suppression of pathogen-associated MGEs, indicating that FF can effectively reduce the risk of ARG dissemination. However, the potential impact of residual antibiotics still warrants attention. Redundancy analysis showed that TC and TN primarily explained bacteriome and resistome variation under RM, whereas pH, EC, AP, and AK were more strongly associated with FF. Co-occurrence analysis further suggested that fertilizer-driven microbial community shifts may regulate ARG persistence and potential cross-ecosystem dissemination. Overall, fermented fertilizer attenuated, but did not eliminate, manure-derived resistance risks in aquaculture sediments. These findings support fermented fertilizer as a safer management option than raw manure and highlight the need for integrated risk assessment combining ARGs, MGEs, microbial hosts, and antibiotic residues.

## 1. Introduction

Aquaculture is the main driver of global fishery product supply growth [1]. It plays a crucial role in animal protein supply and food security [2]. According to FAO SOFIA 2024, the global aquaculture production in 2022 reached 130.9 million tonnes, with the production of aquatic animals accounting for 94.4 million tonnes, or 51% of the global aquatic animal production [3]. With the intensification of aquaculture, antibiotics were once used for disease prevention and treatment, and in some farming systems, they were used as growth promoters [4]. Although the regulation of veterinary antibiotics and feed additives has become increasingly stringent, historical residues, sediment accumulation, and continued inputs into aquaculture may still exert persistent selective pressure for resistance [5]. Consequently, aquaculture environments have evolved into major reservoirs of antibiotic resistance genes (ARGs), posing severe threats to One Health frameworks [6,7,8]. Beyond direct antibiotic inputs, other management practices can also exert selective pressures [9]. Among these practices, pond fertilization is also a routine management practice used to enhance primary productivity and maintain nutrient supply, particularly in semi-intensive pond aquaculture [10]. Fertilization can alter sediment physicochemical properties, including organic matter, total carbon, total nitrogen, and available phosphorus, thereby restructuring microbial community composition [11]. Previous studies have shown that improper fertilization accelerates ARG spread by introducing exogenous resistant bacteria and intensifying nutrient-mediated selective pressures [12,13,14]. These findings highlight fertilization as an important driver of resistome dynamics in aquaculture environments.

Common fertilization practices include raw manure, inorganic fertilizers, and fermented organic fertilizers, which differ markedly in their inputs of exogenous microorganisms, nutrients, ARGs, and MGEs [15,16,17,18]. Raw manure carries a heavy load of gut derived microorganisms and resistance determinants, its direct application may introduce livestock associated ARGs, antibiotics, MGEs, and allochthonous taxa into receiving sediments and increase opportunities for horizontal gene transfer (HGT), making it a recognized high-risk pathway for ARG dissemination [19,20]. Fermented organic fertilizers are frequently advocated as a safer alternative, as thermophilic fermentation effectively eliminates pathogens and markedly reduces ARG abundance at the raw-material stage [18,21,22], but residual ARGs, MGEs, antibiotics, or adapted microbial hosts may persist after treatment. Inorganic fertilizers, by contrast, do not introduce exogenous microorganisms or manure associated ARGs, but it can reshape indigenous microbial communities by modulating physicochemical niches (e.g., pH and nutrient stoichiometry), indirectly selecting for resistant phenotypes [23]. Crucially, however, current biosafety assessments have largely focused on ARG and pathogen reduction during fermentation or in the final fertilizer product, whereas much less is known about the fate of these risks after fermented fertilizers are applied to aquaculture sediments [24]. Once introduced into aquaculture sediments, residual ARGs, MGEs, antibiotics, and microbial hosts may be reshaped by sediment physicochemical conditions, indigenous microbial communities, and aquaculture management practices. Therefore, whether the biosafety benefits achieved during fermentation persist after application remains unclear, highlighting the need to compare raw manure, inorganic fertilizer, and fermented fertilizer under aquaculture production-cycle conditions.

Determining the persistence of these biosafety benefits requires a mechanistic understanding of ARG fate within the receiving environment. The fate of ARGs is largely governed by the dynamics of their bacterial hosts. Accumulating evidence indicates that sediment microbial community structure is a central determinant of resistome behavior [25,26,27]. Fertilization acts as a key exogenous environmental driver that reshapes community structure and alters the ecological niches for pathogenic ARG hosts [28]. We hypothesize that fermented fertilizers may promote a low-risk community succession by altering microbial community structure. Mechanistically, the shift in microbial composition may reduce the abundance of opportunistic pathogens, thereby decreasing the reservoir of host-associated ARGs in aquaculture sediments [29,30]. However, these potential mechanisms have yet to be systematically validated.

Crucially, the presence or abundance of ARGs does not directly equate to health risk [31]. In fact, ARGs are ubiquitous across environments and are widely detected even in relatively pristine ecosystems [32]. Using ARG abundance alone as a proxy for health risk assessment is insufficient; instead, the actual risk is determined by high-risk ARGs that are closely associated with mobile genetic elements (MGEs) [33,34,35]. However, most existing studies do not distinguish between background ARGs and high-risk ARGs, hindering accurate assessment of fertilization-induced ecological impacts [33,36,37]. In particular, the influence of different fertilization strategies on the distribution of high-risk ARGs and their co-occurrence with potential pathogens remains largely unexplored [38,39,40]. This knowledge gap leads to a critical underestimation of biosafety risks, as the reduction in total ARG abundance may mask the selective enrichment of high-risk ARGs.

Based on the above research gaps, a long-term aquaculture experiment was established to systematically compare the effects of fermented fertilizers and conventional fertilization practices on the sediment resistome. The experiment was designed to (1) evaluate whether fermented fertilizers could reduce the composition and abundance of ARGs by modulating microbial community structure and host–ARG co-occurrence patterns, and (2) determine how different fertilization regimes influenced high-risk ARGs, which possess elevated mobility potential and potential pathogenic relevance. By integrating microbial community ecology with resistome analysis, this study aims to provide a new perspective for assessing the ecological safety of organic fertilizers in aquaculture and offers scientific guidance for controlling high-risk ARGs in aquatic environments.

## 2. Materials and Methods

### 2.1. Site Information and Experimental Design

The sampling site located in Hailing District, Taizhou City, Jiangsu Province, China (32°29′ N, 119°54′ E), a region characterized by a typical northern subtropical monsoon climate. The total area of the experimental ponds was 48,000 m^2^, which was subsequently divided into 24 individual ponds of 2000 m^2^ each at the beginning of the experiment. The initial levels of antibiotic residues were assumed to be uniform across all ponds. Three fertilization treatments were established to represent different practical pond management scenarios and potential ARG input pathways: (1) IF (inorganic fertilizers only), ponds received only chemical fertilizers, (2) RM (raw manure), a 3:7 (*w*/*w*) mixture of fresh chicken and pig manure, and (3) FF (fermented organic fertilizers), prepared by fermenting RM with a microbial inoculant (yeast: *Bacillus*: Actinomycetes = 25:50:25) at 55–70 °C for 15 days. Each treatment included 8 replicate ponds. The three treatments were randomly assigned across the 24 ponds, and each pond was equipped with independent inlet and drainage channels to ensure hydrological separation between replicate ponds. Subsequently, *Eriocheir sinensis* (18,000 individuals per hectare) and *Macrobrachium nipponense* (300,000 individuals per hectare) were continuously co-cultured in all ponds. Aside from fertilization type, ponds followed the same routine aquaculture management schedule and stocking regime, and no intentional antibiotic treatment was applied during the 6-month culture period.

Fertilization was conducted as follows: the first application was on 20 February 2024 at a rate of 1200 kg/ha, followed by 15 days applications of 75 kg/ha from 1 April to 30 June 2024.

### 2.2. Pond Sediment Sampling

Following a 6-month culture period, sediment samples were collected from each pond in November 2024. Three sediment cores (2–10 cm depth, excluding the surface sludge layer) were randomly obtained from each pond using a cylindrical sampler and combined into a single composite sample, resulting in 8 samples per treatment. After collection, samples were packaged in airtight sterile plastic bags and transported within icebox. Each sample was divided into two parts: one was stored at 4 °C for immediate determination of chemical properties and antibiotic concentrations, the other was archived at -80 °C for subsequent DNA extraction.

### 2.3. Sediment Properties Determination

Sediment pH and electrical conductivity (EC) were measured via a pH meter and conductivity meter (Thermo Fisher Scientific, Waltham, MA, USA), respectively. Total carbon (TC) and total nitrogen (TN) were quantified using a Vario EL cube elemental analyzer (Elementar Analysensysteme GmbH, Langenselbold, Germany). Available phosphorus (AP) was extracted with sodium bicarbonate and determined by molybdenum blue colorimetry; available potassium (AK) was extracted with ammonium acetate and measured via flame photometry. All the results of chemical properties are presented in Supplementary Table S1.

### 2.4. DNA Extraction

Genomic DNA was extracted from 0.5 g aliquots of each sediment sample using the FastDNA^®^ Spin Kit for Soil (MP Biomedicals, Carlsbad, CA, USA), strictly following the manufacturer’s instructions. DNA quality was evaluated using Qubit 3.0 Fluorometer (Thermo Fisher Scientific, Waltham, MA, USA) and NanoDrop One spectrophotometer (Thermo Fisher Scientific, Waltham, MA, USA) [41].

### 2.5. Sequencing Library Construction and Metagenomic Sequencing

For each sample, approximately 1 μg of genomic DNA was fragmented using a Covaris S220 focused-ultrasonicator (Woburn, MA, USA). Sequencing libraries were prepared using the NEBNext^®^ Ultra™ DNA Library Prep Kit for Illumina^®^ (New England Biolabs, Ipswich, MA, USA) according to the manufacturer’s recommendations. Library size distribution was assessed with a Qsep 400 High-Throughput Nucleic Acid-Protein Analysis System (Hangzhou Houze Biological Technology Co., Ltd., Hangzhou, China), and the concentrations were quantified using the Qubit 3.0 Fluorometer (Thermo Fisher Scientific, Waltham, MA, USA). Finally, metagenomic shotgun sequencing was conducted on the Illumina NovaSeq platform by Guangdong Magigene Biotechnology Co., Ltd. (Guangzhou, China). The raw sequencing data generated in this study have been deposited in the Genome Sequence Archive (GSA) at the National Genomics Data Center (NGDC), China National Center for Bioinformation (CNCB), under accession numbers CRA040658, CRA040666, and CRA040644. A summary of sequencing quality metrics for all shotgun metagenomic samples, including raw reads, raw bases, clean reads, clean bases, Q20, Q30, GC content, and effective rate, is provided in Supplementary Table S3.

### 2.6. Raw Sequencing Data Processing and Taxonomy Profiling

Raw reads were quality-filtered using Trimmomatic (version 0.36) [42]. The high-quality reads were then de novo assembled into contigs using MEGAHIT (version 1.2.9) [43]. All assembled contigs longer than 500 bp were used for open reading frame (ORF) prediction with Prodigal (version 2.6.3) [44]. Taxonomic classification was performed using Kraken2 (version 2.1.3) [45,46] based on k2_standard_202409 database. The classification results were further estimated for the profiling the relative abundance of the taxa using Bracken software (version 2.9) [47,48].

### 2.7. ARG Prediction

ARG profiles were identified from clean reads using the ARG-OAP pipeline (version 3.1) [49]. ARG-like sequences were retained only when meeting all of the following thresholds: BLASTX e-value ≤ 1 × 10^−7^, amino acid identity ≥ 80%, and alignment length ≥ 75%. The retained ARG like sequences were summarized and compared among different samples at the type, subtype, and individual gene levels. The abundance *f* of ARGs was quantified using the Transcripts Per Million (TPM) method to normalize for gene length and sequencing depth, calculated as follows [50]:

$$G_{k}=\frac{r_{k}}{L_{k}}*\frac{1}{\sum_{i=1}^{n}\frac{r_{i}}{L_{i}}}*10^{6}$$

where *r_k_* is the number of reads aligned to the kth gene, *L_k_* is the length of the kth gene, and $\sum_{i=1}^{n}\frac{r_{i}}{L_{i}}$ is the sum of values after normalization of all (*n*) genes by length.

### 2.8. MGEs Prediction

MGE-associated genes were annotated using mobileOG-dp database (https://github.com/clb21565/mobileOG-dp, (accessed on 1 April 2026)) [51], including transposases, insertion sequence (IS) elements, integrases, Tn7-related proteins (istA and istB), transposition proteins (tniA and tniB), and plasmid-associated proteins. Specifically, protein sequences predicted from metagenomic assemblies (Section 2.5) were aligned against the MGE reference database using BLASTP (version 2.15.0) [52]. Alignments were considered significant if they met the following criteria: an e-value ≤ 1 × 10^−7^, amino acid identity ≥ 80%, and alignment coverage ≥ 75%.

### 2.9. Antibiotics Determination

The concentrations of eight commonly used antibiotics, including β-lactams (ampicillin, amoxicillin), tetracyclines (tetracycline, doxycycline), sulfonamides (sulfadimidine, sulfamethoxypyridazine, sulfaquinoxaline), and macrolides (erythromycin) were determined. The determination was performed using high-performance liquid chromatography coupled with tandem mass spectrometry (HPLC–MS/MS, Agilent 6460, Agilent Technologies, Santa Clara, CA, USA). Chromatographic separation was achieved using an Agilent Poroshell 120 EC-C18 column (2.7 μm, 3.0 × 100 mm, Agilent Technologies, Santa Clara, CA, USA). Ground sediment (1.0 g) was extracted with 10 mL of acetonitrile and 2 mL of EDTA-McIlvaine buffer (pH = 4.0). Then, 0.8 g of sodium sulfate and 0.2 g of sodium chloride were added to facilitate salting-out and phase separation, followed by standing for 10 min. After centrifugation at 8000× *g* and 4 °C for 10 min, the supernatant was diluted with ultrapure water to reduce the organic solvent content to less than 10%. Subsequently, the samples were purified and enriched via solid-phase extraction (SPE) using Oasis HLB cartridges (Milford, MA, USA). The cartridges were eluted with methanol, and the eluate was evaporated to near-dryness under a gentle nitrogen stream at 30 °C. The residue was reconstituted in 0.2 mL of the initial mobile phase and filtered through a 0.22 μm organic membrane prior to HPLC–MS/MS analysis. The results of the antibiotic concentrations are listed in Supplementary Table S2.

### 2.10. Statistical Analysis

All statistical analyses were performed using R software (version 4.5.2) [53]. One-way analysis of variance (ANOVA) was used to test differences in sediment physicochemical properties and antibiotic concentrations among the IF, RM, and FF treatments, whereas the Kruskal–Wallis test was applied to evaluate differences in the abundances of major ARGs among treatments, with the significance level set at *p* < 0.05. Stacked bar charts were used to display the composition of dominant taxa, ARGs, and MGEs in sediments under different treatments. Based on Bray–Curtis distances, principal coordinate analysis (PCoA) was performed to assess microbial community composition, resistome structure, and MGE profiles across treatments [54,55], and permutational multivariate analysis of variance (PERMANOVA) was further used to evaluate differences in Beta-diversity among groups [56]. Venn diagrams were used to visualize shared and unique ARG or MGE subtypes among treatments. Redundancy analysis (RDA) was conducted to assess the relationships between sediment physicochemical factors and bacterial community or ARG composition [57].

Co-occurrence networks were constructed to evaluate co-occurrence patterns among microbial communities, MGEs, and ARGs [58]. Based on the relative abundance matrices of microorganisms, ARGs, and MGEs, Spearman’s correlation coefficients were calculated to determine correlations among dominant taxa, ARGs, and MGEs; correlations with |*r*| > 0.8 and FDR adjusted *p* < 0.05 were considered significant [59].

High-risk ARGs were defined as ARGs with potential mobility and pathogenic relevance, following recently proposed ARG risk ranking frameworks [35,36]. ARG risk was evaluated from two dimensions: mobility potential and pathogenic relevance. Mobility potential was inferred from MGE profiles and ARG–MGE co-occurrence patterns, whereas pathogenic relevance was assessed based on co-occurrence associations between ARGs and pathogen-, host-, or disease-associated annotations.

## 3. Results

### 3.1. Fertilization Regimes Significantly Altered Sediment Physicochemical Properties

Sediment physicochemical properties varied significantly across different fertilization treatments (*p* < 0.05, Table S1). The IF treatment exhibited the highest contents of TC, TN, AP, and AK. The FF treatment showed the highest values of pH and EC, but the lowest levels of TC and TN. The RM treatment displayed the lowest values for EC, AP, and AK.

### 3.2. Fertilization-Driven Shifts in Sediment Microbial Community Structure and Composition

**Figure 1:**
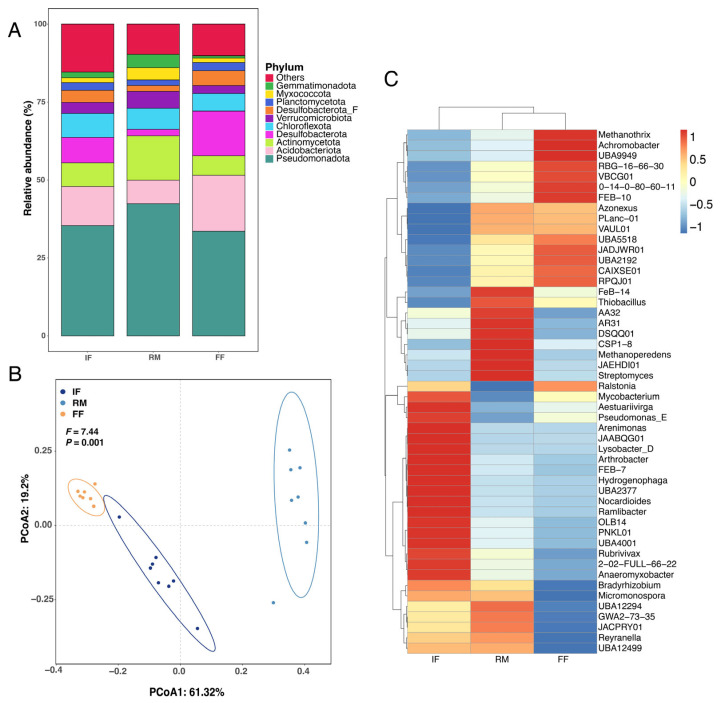
**Microbial community composition and structural shifts.** Stacked bar chart displaying the relative abundance (%) of the main bacterial phyla identified in sediment samples from different treatments (**A**). PCoA plots showing the differences in sediment microbial composition among different treatments based on Bray–Curtis distance (**B**). Heatmap depicting the differences in relative abundance of the top 50 abundant microbial genera among different treatments (**C**).

Taxonomic analysis based on metagenomic sequencing revealed that at the phylum level, the top 10 abundant bacteria phyla in all sediment were *Pseudomonadota*, followed by *Acidobacteriota*, *Actinomycetota*, *Desulfobacterota*, *Chloroflexota*, *Verrucomicrobiota*, *Desulfobacterota_F*, *Planctomycetota*, *Myxococcota*, and *Gemmatimonadota* (Figure 1A). The relative abundances of *Acidobacteriota* and *Desulfobacterota* were higher, whereas *Pseudomonadota* and *Actinomycetota* was lower in the IF and FF groups compared with the RM group (Figure 1A). PCoA based on Bray–Curtis distances showed that the microbial community composition was significantly separated among the treatments (*p* = 0.001, Figure 1B). At the genus level, the heatmap demonstrated that the relative abundances of the top 50 abundant genera obviously differed among the IF, RM, and FF treatments (Figure 1C). The FF group was characterized by elevated abundance of *Methanothrix*. In contrast, RM samples were enriched in *Thiobacillus* and *Methanoperedens*. Meanwhile, IF samples showed higher relative abundance of taxa such as *Pseudomonas_E*, *Arenimonas*, *Lysobacter_D*, *Arthrobacter*, and *Hydrogenophaga* (Figure 1C).

### 3.3. Fertilization Regimes Reshaped the Resistome Structure and Key ARG Determinants

**Figure 2:**
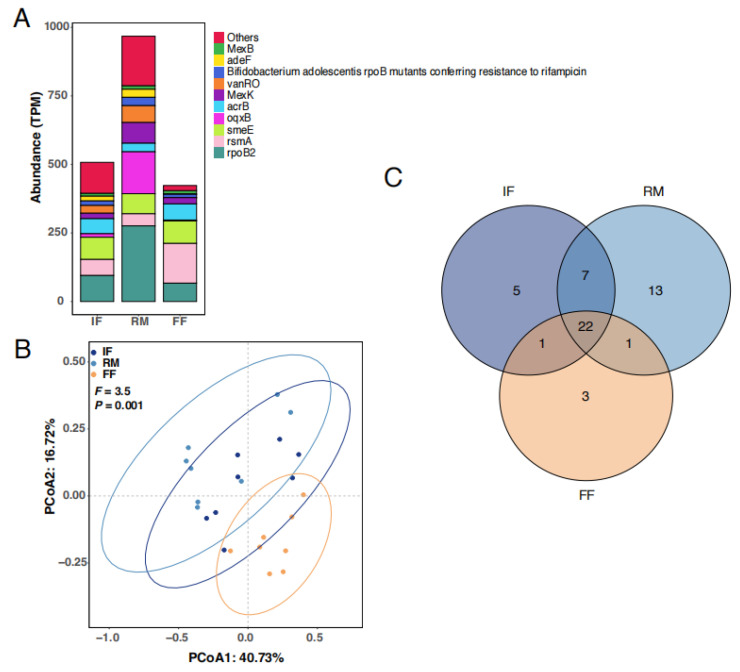
**Quantitative profiling of antibiotic resistance genes (ARGs) and antibiotic concentrations.** Stack bar chart showing the normalized abundance (TPM) of ARGtypes in different treatments (**A**). PCoA plots showing the composition of ARG subtypes among different samples based on the Bray–Curtis distance matrix (**B**). Venn diagram displaying the shared and unique ARGs among different treatments (**C**).

As shown in Figure 2A, the total abundance of ARGs was markedly higher in the RM treatment compared to the IF and FF groups. While the RNA polymerase subunit gene *rpoB2*, *rsmA*, and *smeE* were the dominant subtypes across all treatments. PCoA based on Bray–Curtis distances revealed that the resistome structures were significantly different among the treatments (*p* = 0.001, Figure 2B). Venn diagram analysis showed that a core resistome consisting of 22 ARGs subtypes were shared among all three fertilization treatments (Figure 2C). The RM group exhibited the highest diversity of unique subtypes, with 13 identified, whereas the IF and FF groups contained 5 and 3 subtypes, respectively. Furthermore, the RM group shared seven exclusive subtypes with the IF group, compared with one shared with the FF group.

### 3.4. Variations in ARGs and Antibiotic Residues Under Different Fertilization Regimes

**Figure 3:**
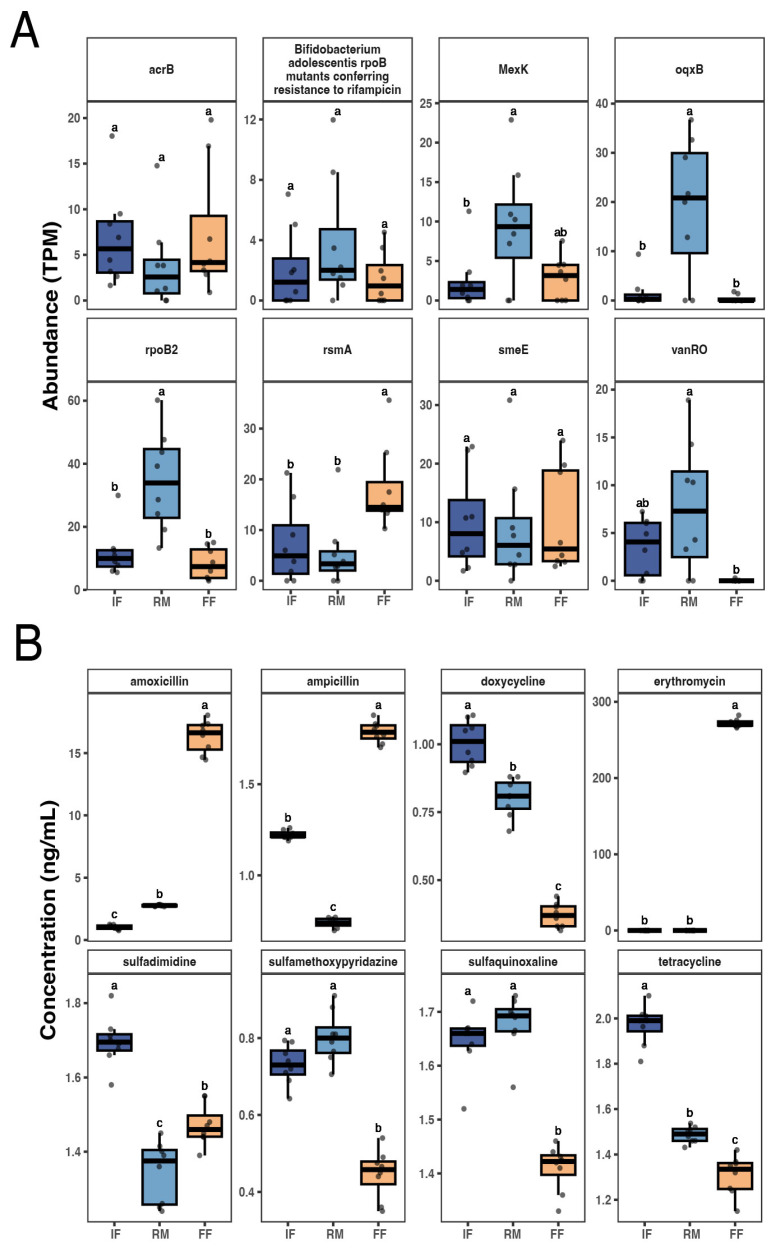
**Quantitative profiling of antibiotic resistance genes (ARGs) and antibiotic concentrations across the IF, RM, and FF groups.** Boxplot showing the abundance of major ARGs in different samples (**A**). Different letters above the histogram indicate significant differences at *p* < 0.05 level. Concentrations of detected antibiotic residues in sediments treated with different fertilizer treatments (**B**). Different lowercase letters (a, b, c) indicate significant differences between groups at *p* < 0.05, while the same letter indicates no significant difference.

We further quantified the abundance of specific ARGs and the concentration of antibiotic residues to evaluate the potential risks associated with different fertilization treatments. The profiles of ARGs varied significantly among the groups (Figure 3A). The RM treatment significantly increased the abundance of multidrug resistance genes, such as *MexK*, *oqxB*, *rpoB2* and *vanRO*, compared to the IF and FF groups (*p* < 0.05). In contrast, the FF group exhibited significantly lower abundances of these genes but showed a specific enrichment of *rsmA*. Other genes, including *acrB* and *smeE*, showed no significant differences across treatments. These results suggested that although the FF group reduced the load of certain ARGs like *oqxB*, it did not eliminate all antibiotic residues, particularly β-lactams and macrolides.

### 3.5. Distinct Fertilization Regimes Led to Divergent Antibiotic Residue Profiles in Sediments

The concentrations of antibiotic residues also displayed distinct patterns (Figure 3B, Table S2). Interestingly, the FF group contained the highest concentrations of β-lactams (amoxicillin and ampicillin) and the macrolide erythromycin. Conversely, the IF group was characterized by the highest levels of tetracyclines (doxycycline and tetracycline) and the sulfonamide sulfadimidine, with all concentrations significantly exceeding those in the RM and FF groups (*p* < 0.05). For sulfamethoxypyridazine and sulfaquinoxaline, IF and RM maintained relatively higher concentrations, whereas FF was significantly lower (*p* < 0.05).

### 3.6. Differential Enrichment of MGEs Indicates Altered Mobility-Related Resistance Risks

**Figure 4:**
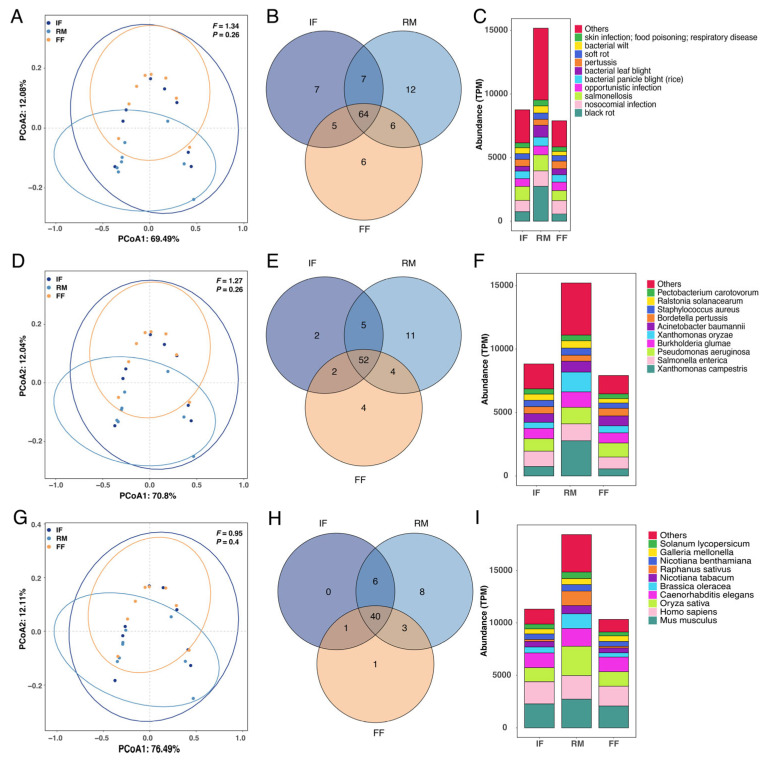
**Multi-level characterization of potential pathogenic bacterial communities.** Comprehensive analysis of MGE subtypes across three categorization schemes. The rows represent MGE data categorized by disease (**A**–**C**), pathogen (**D**–**F**), and host (**G**–**I**), respectively. Left column (**A**,**D**,**G**): PCoA plots showing MGE subtype compositions based on Bray–Curtis distances. Middle column (**B**,**E**,**H**): Venn diagrams highlighting shared and unique MGE subtypes among treatments. Right column (**C**,**F**,**I**): Stacked bar charts depicting the TPM-normalized abundance of MGE types.

PCoA revealed no statistically significant differences in the overall community structure of MGEs among the three fertilization treatments across all classification categories (*p* > 0.05, Figure 4A,D,G). Venn diagrams were utilized to disentangle the shared and unique MGE subtypes associated with specific diseases, pathogens, and hosts (Figure 4B,E,H). A core set of MGEs was shared among all treatments, including 64 disease-associated, 52 pathogen-associated, and 40 host-associated subtypes. The RM treatment introduced a disproportionately high number of unique subtypes. Specifically, the RM group harbored 12 unique disease-associated subtypes, 11 unique pathogen-associated subtypes, and 8 unique host-associated subtypes, significantly outnumbering those in the IF and FF groups.

The RM treatment exhibited a markedly higher total abundance of MGEs compared to the IF and FF groups (Figure 4C,F,I). The total abundance of MGEs in the RM-treated sediments reached approximately 15,000 TPM across all classification categories, representing a nearly two-fold increase compared to the IF and FF treatments. Specifically, the RM group was characterized by a marked enrichment of MGEs associated with black rot and nosocomial infections, which were significantly less abundant in the IF and FF groups (Figure 4C). The surge in MGE abundance in the RM group was primarily attributed to elements linked to *Xanthomonas campestris* and *Xanthomonas oryzae*. In contrast, the FF treatment effectively suppressed the abundance of these pathogen-associated MGEs (Figure 4F). Crucially, the RM treatment showed a dramatic accumulation of MGEs associated with diverse hosts (Figure 4I). In addition to mammalian hosts such as *Mus musculus* and *Homo sapiens*, MGEs associated with economically important crops, specifically *Oryza sativa*, *Brassica oleracea*, and *Raphanus sativus*, were noticeably enriched in the RM group compared to the IF and FF treatments.

### 3.7. Environmental Factors Differentially Governed Microbial Community Assembly and Resistome Variation

**Figure 5:**
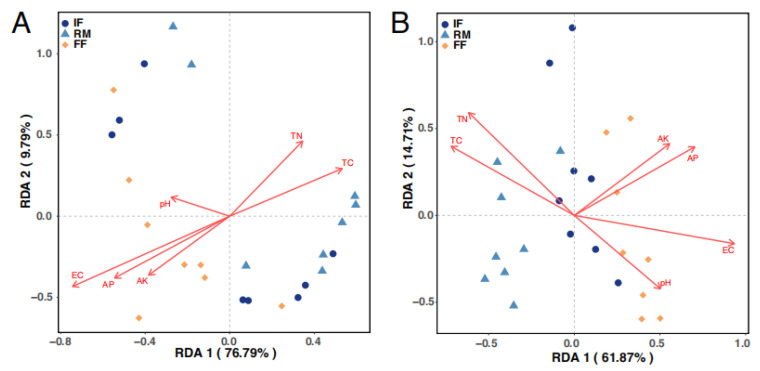
**Redundancy analysis (RDA) revealing the relationships between environmental factors and biological profiles.** Distance-based redundancy analysis (RDA) showing the correlations between bacterial communities (**A**) or antibiotic resistance gene (ARG) types (**B**) and sediment chemical properties in different fertilized samples. Sediment properties (red arrows): EC, electrical conductivity; pH; TC, total carbon; TN, total nitrogen; AP, available phosphorus; AK, available potassium.

The RDA illustrated that the components of RDA1 and RDA2 explained 86.58% of the total bacterial variations (Figure 5A) and 76.58% of the ARGs variations (Figure 5B). These high explanatory values indicated that the measured sediment chemical properties including pH, TC, TN, AP, AK, and EC explained the dominating variation in determining the composition of bacterial community and ARGs. In the bacterial community analysis (Figure 5A), the RM treatment was correlated with TN and TC, indicating that organic carbon and nitrogen inputs were the primary determinants for the bacteriome in the raw manure group. Conversely, the FF treatment clustered closely with AP, AK, and EC, suggesting that the availability of dissolved nutrients and salinity drove the bacterial succession in the fermented fertilizer group. In contrast, the IF group was situated away from the vectors of TC, TN, AP, AK, and pH. A consistent pattern was observed in the RDA of ARG types (Figure 5B). The distribution of ARGs in the RM group was strongly associated with TN and TC vectors. Similarly, the ARGs in the FF group showed a strong correlation with EC, AP, AK and pH.

### 3.8. Co-Occurrence Network Analysis Reveals Potential Associations Among ARGs, Microbial Taxa, and Environmental Factors

**Figure 6:**
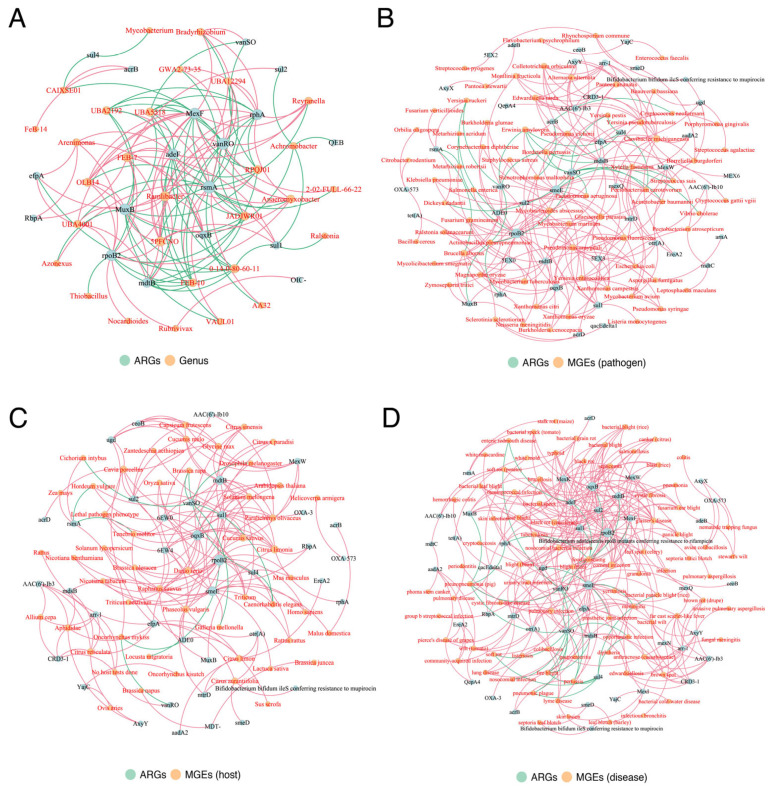
**Network interactions between ARGs, Mobile genetic elements (MGEs), and microbial genera.** Network diagrams illustrate the associations of major ARG subtypes with microbial genera (**A**) and MGEs (**B**–**D**). The connections are based on significant Spearman’s correlations (|*r*| > 0.8, *p* < 0.05). Nodes are scaled by their total number of significant connections (degree), with red edges indicating positive correlations and green edges indicating negative correlations.

The co-occurrence network at the genus level (142 edges, 47 nodes) showed significant correlations in the co-occurrence patterns between ARG subtypes and microbial taxa (Figure 6A). Specifically, *Ramlibacter* was significantly and positively correlated to the multidrug resistance genes (*mdtB*, *MexF* and *adeF*), vancomycin resistance gene *vanRO*, rifampicin resistance gene *rpoB2*, and quinolone resistance gene *oqxB*. *Reyranella* showed significant positive correlations with *vanRO*, rifamycin resistance gene *rphA*, and multidrug resistance genes (*MexF*, *MuxB* and *adeF*). *Arenimonas* showed robust positive correlations with *rpoB2* and *oqxB*. On the contrary, *Achromobacter* exhibited broad and significant negative correlations with sulfonamide resistance genes (*sul1* and *sul2*) and *oqxB*.

The pathogen-ARG network (228 edges, 114 nodes) highlighted the potential health risks and carriage of resistance genes by pathogen (Figure 6B). Notably, for *Brucella abortus*, a significant zoonotic pathogen, its relative abundance showed significant positive correlations with *vanRO*, *MexF*, *MuxB*, *adeF*, and *mdtB*. *Glaesserella parasuis* was significantly and positively correlated with *sul1*, *sul2*, *MexK*, and *MexW*. *Pseudomonas aeruginosa*, *Erwinia amylovora* and *Pectobacterium carotovorum* were associated with the multidrug efflux gene *smeE*, showing an significant positive correlation. *Stenotrophomonas maltophilia* displayed significant positive correlations with *oqxB* and *MexK*. Furthermore, the *rpoB2* was significantly conserved within the *Mycobacterium* genus, showing strong positive correlations with both *Mycobacterium tuberculosis* and *Mycobacterium marinum*.

The host tracking network (173 edges, 89 nodes) was used to explore the dissemination of ARGs across the eukaryotic interfaces (Figure 6C). Mammalian hosts, including *Homo sapiens* and *Mus musculus* showed significant positive correlations with *smeE* and *rpoB2*. In the aquaculture sector, the model organism *Danio rerio* was strongly associated with *sul1* and *oqxB*. *Oncorhynchus mykiss* exhibited significant negative correlations with *adeF* and *vanRO*. Additionally, agricultural crops such as *Oryza sativa* were significantly correlated with *rpoB2* and *smeE*.

The disease-association network (315 edges, 134 nodes) elucidated the potential clinical consequences of the detected ARGs (Figure 6D). Cystic fibrosis was significantly and positively correlated with *sul2*, *oqxB*, *MexK* and *adeF*. Brucellosis was characterized by strong associations with *MuxB* and *rphA*. *SmeE* was strongly linked to severe infections, including nosocomial infection, opportunistic infection, and pneumonia. Consistent with the pathogen analysis, *rpoB2* showed a specific and high-confidence correlation with tuberculosis. In the context of plant pathology, Fire blight displayed a highly specific and strong positive correlation with *smeE*, while showing significant negative correlations with vancomycin resistance genes (*vanSO* and *vanRO*). Black rot showed robust positive correlations with *oqxB*, *rpoB2* and *sul1*. Furthermore, Bacterial blight was significantly correlated with *sul1* and *rpoB2*.

## 4. Discussion

Microorganisms have been widely reported as important reservoirs of ARGs [60]. The introduction of ARG-hosting bacteria through fertilization can alter the local microbial communities, influencing ARG proliferation and reduction [61]. Previous studies have demonstrated strong correlations between ARG profiles and bacterial community composition in soils [62], sediments [63], and aquatic environments [64]. Consistent with these findings, similar patterns were observed in aquaculture sediments in this study (Figure 5A) [60]. More importantly, our results indicate that this coupling phenomenon is driven by environmental factors (Figure 5B). Specifically, After RM treatment, the sediment exhibited the high levels of total carbon and total nitrogen, along with the highest abundance of ARGs (Table S1, Figure 2A). Total carbon and total nitrogen serve as critical nutrients, stimulating microbial growth and metabolic activities [65,66]. Enhanced microbial biomass may intensify interspecific competition, driving bacteria to acquire ARGs via horizontal gene transfer mechanisms such as plasmid conjugation or transposon-mediated transfer [34,67,68]. Under such conditions, the sediment environment likely supported both the persistence of manure-derived resistant populations and the secondary proliferation of indigenous ARG-bearing taxa [69]. In contrast, high-temperature fermentation in the FF treatment likely transformed part of the original manure organic matter into more mineralized or bioavailable forms, thereby shifting the dominant environmental drivers toward readily available nutrients (AP, AK), pH, and salinity (EC) (Table S1). This suggests that FF treatment reduced nutrient loading and changed the quality and accessibility of sediment resources, together with ionic and acid–base conditions. Salinity is a crucial determinant of microbial community composition, mainly by increasing the selective pressure on microorganisms, which might consequently influence the proliferation of ARGs [70]. Serrana et al. [71] also observed that the environmental resistome strongly correlates with the salinity gradient, and the diversity of ARGs in sediments declining with increasing salinity. Similarly, pH may indirectly affect ARGs through the proton motive potential, which drives the activity of multidrug efflux pumps [72]. Efflux pump-associated transporters often exhibit promiscuity, which enables them to transport multiple antibiotics [73]. Therefore, the transmission, persistence, and enrichment of ARGs in natural ecosystems are ecological processes jointly driven by both biotic and abiotic factors. [74]. The observed resistome differentiation among fertilization treatments is not an isolated genetic response, but rather a downstream outcome of sediment physicochemical regulation on host community assembly. Therefore, physicochemical conditions should be regarded as the prerequisite for resistome divergence.

The application of raw manure markedly increased the risk of ARG dissemination in the environment [75]. Specifically, the RM treatment exhibited the highest total ARG abundance and the greatest number of unique ARG subtypes, along with a significant enrichment of multiple multidrug resistance genes, including *MexK*, *oqxB*, and *vanRO* (Figure 2A,C). These findings indicate that untreated manure inputs not only expand the environmental resistome but also increase its structural complexity [76]. Notably, the elevated risk associated with RM cannot be explained solely by ARG abundance, but is strongly linked to enhanced mobility and ecological connectivity of resistance genes. MGEs are key drivers of gene transfer, among which MGE-mediated horizontal gene transfer represents the primary mechanism underlying the dissemination of ARGs in sediment ecosystems [60,77,78]. Our results consistently showed that RM treatment significantly increased both the abundance and diversity of MGEs, particularly those associated with pathogen categories (Figure 4). For instance, MGEs linked to plant pathogens responsible for black rot (*Xanthomonas campestris*) and bacterial blight (*Xanthomonas oryzae*), as well as human-associated pathogens involved in nosocomial infections, were markedly enriched. Furthermore, host-associated MGEs exhibited expanded associations with a wide range of organisms, including *Homo sapiens*, *Mus musculus*, and economically important crops such as *Oryza sativa* and *Brassica oleracea*, highlighting a broader cross-kingdom dissemination potential. These findings further support the framework proposed by Zhang et al. (2021), which emphasizes that antimicrobial resistance risk should be assessed by integrating ARG abundance, accessibility, potential contribution to pathogenicity, mobility between hosts and relevance to clinical treatment [79]. Within this framework, raw manure application not only increases the ARG load but also is associated with elevated MGE co-occurrence signals, thereby shifting the resistome toward a more mobile and clinically relevant configuration. This implies that the direct application of untreated livestock manure into aquaculture systems effectively transfers livestock-associated resistomes into aquatic environments. This process may correspond to elevated mobility-related risk signals and supports the view that livestock manure serves as a global reservoir of clinically relevant resistance genes [76].

Compared with RM, FF showed lower ARG abundance, fewer unique ARG subtypes, and reduced levels of several risk-relevant genes, including *MexK*, *oqxB*, *rpoB2*, and *vanRO* (Figure 2A,C). FF also showed lower pathogen-associated MGE signals than RM, indicating that fermentation pretreatment effectively reduced the risk of manure-derived resistome in aquaculture sediments (Figure 4). This pattern is consistent with previous studies showing that thermophilic composting or fermentation can reduce manure-borne ARGs, MGEs, and pathogenic microorganisms, although residual resistance risks may persist in treated manure products [80]. The mitigation observed in FF may be explained by processes occurring during both fermentation and post application stages. During thermophilic pretreatment, elevated temperature and microbial turnover likely reduced manure-derived resistant hosts and MGEs, thereby weakening pre-existing ARG–MGE–host linkages before fertilizer application. Nevertheless, some ARG–MGE associations may persist in surviving or adapted microbial populations. After FF was introduced into aquaculture sediments, these residual associations could be further filtered and reorganized by sediment physicochemical conditions and indigenous microbial communities [80]. Because bacterial community composition is closely linked to resistome structure in soils, sediments, and aquatic environments, these post-application changes in sediment conditions may have contributed to the distinct microbial and ARG profiles observed in FF-treated sediments [62,64]. Importantly, the lower ARG signals in FF did not correspond to uniform reductions in all antibiotic residues, revealing a decoupling between antibiotic residue concentrations and ARG abundance. In particular, β-lactam antibiotics, including amoxicillin and ampicillin, and the macrolide erythromycin remained relatively high in FF-treated sediments (Figure 3B). The elevated β-lactam residues in FF-treated sediments may reflect incomplete thermal degradation during fermentation, differential partitioning of specific compounds into sediment organic matter, or pre-existing inputs from feed, water, or pond history; however, the exact source cannot be resolved from the present data. Previous studies have shown that antibiotic attenuation during manure treatment is compound-specific and can be affected by organic matrices, sorption, and transformation processes [81,82]. Therefore, FF should be interpreted as a risk-reduction strategy relative to RM, rather than a risk-free amendment. The specific enrichment of *rsmA* in FF further suggests that individual ARG subtypes may persist or respond differently from the total resistome (Figure 3A). Such subtype-specific persistence could be related to adapted microbial hosts or local selection under the altered sediment environment. Functionally, these residual ARGs may act as a latent resistance reservoir in FF treated sediments. If expressed by viable hosts or associated with MGEs, they could contribute to microbial tolerance under renewed antibiotic or environmental stress and may increase the potential for horizontal transfer within sediment microbial communities [74,77]. However, because this study did not resolve contig-level ARG–MGE–host linkages, the enrichment of *rsmA* should not be interpreted as direct evidence of enhanced mobility. Future work should characterize ARGs, MGEs, antibiotic residues, and microbial hosts in raw manure and fermented fertilizer before application, and should incorporate assembly-based ARG–MGE co-localization, metagenomic binning, or long-read sequencing to determine whether persistent ARGs are physically linked to MGEs or specific hosts.

Co-occurrence network analysis revealed the potential ecological and clinical implications of resistome dissemination in aquaculture sediments. Bacteria are the major reservoirs, hosts, and potential vectors of ARGs in the environment. An individual ARG is often carried by multiple potential bacterial hosts [83]. Meanwhile, bacteria harboring multiple resistance genes may exhibit resistance to a wide range of antibiotics [84]. Previous studies have shown that Proteobacteria are major carriers of ARGs [85,86]. Consistent with these findings, our microbial host-association network also revealed that certain taxa within Proteobacteria (e.g., *Reyranella* and *Arenimonas*) were significantly associated with the co-occurrence patterns of ARGs, including *vanRO*, *rpoB2*, and *oqxB* (Figure 6A). Notably, the significant negative correlations of *Achromobacter* with *sul1*, *sul2*, and *oqxB* may reflect antagonistic or competitive interactions within the sediment microecosystem (Figure 6A). Given the strong capacity of *Achromobacter* to degrade organic pollutants and tolerate high-salinity stress, it could potentially suppress sensitive taxa harboring these ARGs through efficient niche occupation or the secretion of secondary metabolites [87,88]. This apparent “host suppression” pattern implies a potential biocontrol strategy for mitigating resistance dissemination through the regulation of indigenous functional microbial communities. The pathogen-associated network further highlighted the linkage between the sediment resistome and potential health risks. For example, positive correlations were observed between *smeE* and *Pseudomonas aeruginosa*, *Erwinia amylovora*, and *Pectobacterium carotovorum*, indicating that this gene may co-occur with taxa spanning both opportunistic human pathogens and phytopathogens in aquaculture sediments (Figure 6B). In the aquaculture context, the co-occurrence between *rpoB2* and *Mycobacterium* is particularly noteworthy (Figure 6B). *Mycobacterium marinum* is a recognized fish pathogen and zoonotic agent, suggesting that resistance signatures in sediments may be relevant to both fish health and human health [89,90]. The host-tracking network further demonstrated that sediment ARGs exhibit broad co-occurrence patterns across eukaryotic reference genomes, involving mammals, aquaculture-associated vertebrates, and crops, thereby providing preliminary ecological evidence consistent with a One Health perspective (Figure 6C). The significant positive correlations of *smeE* with humans and major crops are consistent with a potential exposure pathway, through which ARGs could potentially be transferred across boundaries via the chain of “aquaculture sediment–water/irrigation–agricultural products–human exposure.” Due to ecological barriers, phylogenetic constraints, and limited mobility, these correlations do not confirm direct transfer events, but suggest elevated co-occurrence signals across ecosystem compartments [91,92]. This interpretation was further supported by the disease-association network, which associated environmental ARGs with disease-related annotations (Figure 6D). For instance, the close association of *smeE* with nosocomial infections, opportunistic infections, and pneumonia is consistent with the possibility that the detected resistome contains signature markers relevant to human disease. Meanwhile, the positive correlations of *oqxB*, *rpoB2*, and *sul1* with black rot and bacterial blight indicate that the sediment environment may also harbor resistance genes associated with plant diseases. MGE-driven co-occurrence relationships reinforce the potential penetrative capacity of ARGs of clinical importance in the environment [93]. From a One Health perspective, aquaculture environments are not only sites of food production, but also critical interfaces where environmental microorganisms and human pathogens may exchange ARGs. This cross-ecosystem connectivity underscores the necessity for systematic monitoring and risk assessment of antimicrobial resistance in aquaculture environments.

Fertilization strategies profoundly influence the biosafety landscape of aquaculture systems. Not all organic inputs should be considered inherently safe. Direct application of raw manure should be carefully controlled in aquaculture systems. Although fermented manure (FF) represents a more sustainable alternative, the potential accumulation of specific antibiotic residues warrants careful attention. It is therefore recommended to establish integrated monitoring frameworks that incorporate both chemical residues and biological effects. Future studies should extend the present pond-level replicated design across multiple seasons, sites, and culture cycles, while integrating metatranscriptomic or long-read metagenomic approaches, in order to validate the risk mitigation effects of fermented fertilizers and to further elucidate the mechanistic basis for the persistence and dissemination of ARGs.

Several limitations of the present study should be acknowledged. First, baseline ARG and antibiotic profiles of the sediments before fertilization, as well as the nutrient composition, moisture content, organic matter content, and baseline ARG/antibiotic profiles of the raw manure and fermented fertilizer materials themselves, were not characterized. Consequently, the relative contributions of fertilizer-derived ARG inputs and pre-existing sediment ARG reservoirs cannot be fully partitioned, and the treatment-level differences observed here should be interpreted as the combined outcome of fertilizer composition and post-application environmental selection by sediment physicochemical conditions. Second, the experiment captured a single 6-month culture cycle and does not resolve seasonal or inter-annual dynamics of the sediment resistome. Third, ARG–MGE–host physical linkages were not resolved at the contig or genome level, and the co-occurrence patterns reported here represent statistical associations rather than direct biological linkages.

## 5. Conclusions

In conclusion, long-term fertilization strategy strongly shaped sediment physicochemical conditions, microbial community assembly, resistome composition, and mobility-related risk in aquaculture ponds. Among the three treatments, raw manure posed the greatest biosafety concern by increasing ARG abundance and diversity, enriching multidrug resistance genes, and amplifying pathogen-, disease-, and host-associated MGEs, suggesting a more mobile and ecologically connected resistome. Under the conditions of this 6-month crab–shrimp co-culture pond experiment, fermented fertilizer substantially alleviated manure-derived resistome risks, supporting fermentation as a useful pretreatment strategy before organic fertilizer is applied to aquaculture ponds. Extrapolation of this recommendation to other aquaculture systems, longer time scales, or different fertilizer formulations should be made with caution and require further verification. Moreover, the persistence of specific antibiotic residues and the relative enrichment of individual ARGs indicate that fermentation reduces rather than eliminates resistance risk. Therefore, safer fertilization management in aquaculture should prioritize the pretreatment of organic inputs and adopt integrated monitoring frameworks that jointly evaluate ARGs, MGEs, microbial hosts, and antibiotic residues within a One Health perspective.

## Data Availability

The raw sequencing data generated in this study have been deposited in the Genome Sequence Archive (GSA) hosted by the National Genomics Data Center (NGDC), China National Center for Bioinformation (CNCB), with the accession numbers CRA040658, CRA040666, and CRA040644. These data are publicly accessible via the GSA database (https://ngdc.cncb.ac.cn/gsa/ (accessed on 23 April 2026)). All other relevant data supporting the findings of this study are included in the article or available from the corresponding authors upon reasonable request.

## Supplementary Materials

The following supporting information can be downloaded at: https://www.mdpi.com/article/10.3390/microorganisms14061193/s1, Table S1: Chemical properties of the sampled sediments are presented as mean values (n = 8) ± standard deviation (SD). Table S2: Antibiotic residues of the sampled soils are presented as mean values (n = 8) ± standard deviation (SD). Table S3: Sequencing quality summary of raw and quality-filtered shotgun metagenomic datasets from aquaculture sediment samples under inorganic fertilizer, raw manure, and fermented fertilizer treatments.
