# Supplementary material for: Long-Term Application of Fermented Fertilizer Attenuates the Accumulation of Antibiotic Resistance Genes in Aquaculture Sediment
Source: Microorganisms. 2026 May 25;14(6):1193. doi: 10.3390/microorganisms14061193 (PMC13303721; doi:10.3390/microorganisms14061193)
Supplement: Supplementary file 1 [file microorganisms-14-01193-s001.zip › Table S3 Sequencing quality summary.pdf]

Table S3. Sequencing quality summary of raw and quality-filtered shotgun metagenomic datasets from aquaculture sediment samples under inorganic fertilizer, raw manure, and fermented fertilizer treatments.

Sample ID	Treatment	Raw Reads (M)	Raw Bases (Gb)	Clean Reads (M)	Clean Bases (Gb)	Q20 (%)	Q30 (%)	GC (%)	Effective Rate (%)
RM1	RM	42.36	12.71	40.91	12.07	98.42	95.18	43.21	96.58
RM2	RM	38.74	11.62	37.28	11.00	98.15	94.72	44.85	96.23
RM3	RM	45.83	13.75	44.21	13.04	98.67	95.49	42.67	96.47
RM4	RM	41.29	12.39	39.85	11.76	98.31	95.04	45.32	96.51
RM5	RM	39.62	11.89	38.04	11.22	97.89	94.38	43.78	96.01
RM6	RM	47.18	14.15	45.62	13.46	98.74	95.62	44.12	96.69
RM7	RM	43.55	13.06	41.98	12.38	98.27	94.85	45.91	96.39
RM8	RM	40.18	12.05	38.79	11.44	98.05	94.52	43.55	96.54
IF1	IF	44.62	13.39	43.07	12.71	98.51	95.28	47.32	96.53
IF2	IF	41.95	12.59	40.32	11.89	98.23	94.81	48.15	96.11
IF3	IF	46.71	14.01	45.18	13.33	98.69	95.55	46.84	96.72
IF4	IF	39.48	11.84	37.91	11.18	97.92	94.27	49.21	96.02
IF5	IF	43.27	12.98	41.78	12.33	98.34	95.11	47.65	96.56
IF6	IF	45.84	13.75	44.31	13.07	98.58	95.39	48.42	96.66
IF7	IF	42.16	12.65	40.62	11.98	98.18	94.92	47.08	96.35
IF8	IF	44.93	13.48	43.41	12.81	98.44	95.22	48.73	96.62
FF1	FF	42.85	12.86	41.32	12.19	98.27	95.04	48.85	96.43
FF2	FF	46.32	13.90	44.85	13.23	98.71	95.58	49.73	96.83
FF3	FF	38.96	11.69	37.41	11.04	97.85	94.31	47.62	96.02
FF4	FF	44.71	13.41	43.18	12.74	98.49	95.27	50.34	96.58
FF5	FF	41.58	12.47	40.05	11.81	98.21	94.88	49.12	96.32
FF6	FF	39.27	11.78	37.74	11.13	97.95	94.45	48.27	96.10
FF7	FF	43.84	13.15	42.31	12.48	98.36	95.15	49.58	96.51
FF8	FF	45.67	13.70	44.18	13.03	98.62	95.45	50.18	96.74

Note: Raw bases were estimated from the number of raw read pairs generated by PE150 sequencing. Clean bases represent the retained high-quality sequence data after quality filtering and adapter trimming, and therefore may be slightly lower than raw bases. The effective rate was calculated as the proportion of clean reads relative to raw reads: Clean Reads / Raw Reads × 100%.
