# Supplementary material for: Long-Term Application of Fermented Fertilizer Attenuates the Accumulation of Antibiotic Resistance Genes in Aquaculture Sediment
Source: Microorganisms. 2026 May 25;14(6):1193. doi: 10.3390/microorganisms14061193 (PMC13303721; doi:10.3390/microorganisms14061193)
Supplement: Supplementary file 1 [file microorganisms-14-01193-s001.zip › Table S2.pdf]

Table S2. Antibiotic residues of the sampled soils are presented as mean values (n = 8) ± standard deviation (SD). Different letters indicate significant differences ($P < 0.05$) in mean values among treatments for each chemical property.

Antibiotics (ng/g)	IF	RM	FF
ampicillin	0.24 ± 0.004^{b}	0.14 ± 0.007^{c}	0.35 ± 0.012^{a}
amoxicillin	0.20 ± 0.035^{c}	0.54 ± 0.012^{b}	3.21 ± 0.255^{a}
tetracycline	0.39 ± 0.020^{a}	0.29 ± 0.010^{b}	0.26 ± 0.019^{c}
doxycycline	0.20 ± 0.017^{a}	0.16 ± 0.013^{b}	0.08 ± 0.009^{c}
sulfadimidine	0.33 ± 0.015^{a}	0.26 ± 0.014^{c}	0.29 ± 0.015^{b}
sulfamethoxypyridazine	0.14 ± 0.013^{b}	0.16 ± 0.013^{a}	0.09 ± 0.012^{c}
sulfaquinoxaline	0.32 ± 0.012^{a}	0.33 ± 0.014^{a}	0.28 ± 0.010^{b}
erythromycin	0 ± 0^{b}	0 ± 0^{b}	53.49 ± 1.399^{a}
