# Supplementary material for: Long-Term Application of Fermented Fertilizer Attenuates the Accumulation of Antibiotic Resistance Genes in Aquaculture Sediment
Source: Microorganisms. 2026 May 25;14(6):1193. doi: 10.3390/microorganisms14061193 (PMC13303721; doi:10.3390/microorganisms14061193)
Supplement: Supplementary file 1 [file microorganisms-14-01193-s001.zip › Table S1.pdf]

Table S1. Chemical properties of the sampled sediments are presented as mean values (n = 8) ± standard deviation (SD). Different letters indicate significant differences ($P < 0.05$) in mean values among treatments for each chemical property. Abbreviations: EC, electrical conductivity; TN, total nitrogen; TC, total carbon; AP, available phosphorus; AK, available potassium.

Items	IF	RM	FF
pH	7.72 ± 0.012[b]	7.84 ± 0.342[b]	8.17 ± 0.011[a]
EC (μs/cm)	381.38 ± 3.926[b]	231.13 ± 3.563[c]	841.25 ± 5.007[a]
TC (g/kg)	8.62 ± 0.106[a]	8.45 ± 0.130[b]	7.49 ± 0.121[c]
TN (g/kg)	0.93 ± 0.022[a]	0.86 ± 0.032[b]	0.70 ± 0.036[c]
AP (mg/kg)	30.16 ± 0.998[a]	14.53 ± 0.615[c]	25.17 ± 0.906[b]
AK (mg/kg)	285.42 ± 6.898[a]	143.99 ± 5.511[c]	214.93 ± 4.497[b]
